# Determining the Cause of Recurrent Miscarriages in a Couple: Importance of NOR in the Era of NGS

**Published:** 2019

**Authors:** Usha R. Dutta, Venugopala Swamy, Rajitha Ponnala, Shagun Aggarwal, Ashwin Dalal

**Affiliations:** 1- Diagnostics Division, Center for DNA Fingerprinting and Diagnostics, Hyderabad, India; 2- Department of Medical Genetics, Nizam’s Institute of Medical Sciences, Hyderabad, India

**Keywords:** BAC clones, Bad obstetric history, FISH, Nucleolar organizing regions, Satellites

## Abstract

**Background:**

Chromosomal abnormalities are a significant cause of human disorders. The characterization of such abnormalities helps in the identification of known/ new genes. The purpose of the present study was to identify the cause of miscarriages in a couple by using combined molecular and cytogenetic techniques.

**Case Presentation:**

In this study, the clinical, cytogenetic and molecular cytogenetic evaluations were performed on a couple with recurrent miscarriages. Several methods like GTG banding, silver nitrate (NOR) staining, fluorescence in-situ hybridization (FISH) using whole chromosome paint probes (WCP) and bacterial artificial chromosome (BAC) clones were used. The chromosomal analysis on the metaphases revealed a karyotype of 46,XX in the wife and 46,XY,13p+ in her husband. To check the satellites on 13p region, NOR was performed which showed absence of satellites and presence of euchromatic material. On careful analysis, the satellites were observed on 11q terminal region. Thus, a balanced reciprocal translocation was detected which was confirmed by WCP and Acro-P-arm FISH. Fine mapping with BAC clones narrowed down the breakpoint regions.

**Conclusion:**

The application of the combined cytogenetic methods especially NOR helped in identification of the balanced reciprocal translocation with subsequent systematic characterization and the breakpoint regions were identified. The characterization of the breakpoint regions helped in identification of the carrier status which further paved the way for understanding the cause of recurrent miscarriages and proper genetic counseling.

## Introduction

Recurrent miscarriage (RM) is defined as a condition of three or more consecutive pregnancy losses before 24 weeks of gestation (1). The etiology is unknown in 50% of cases. The causes of RM are parental chromosomal abnormalities, uterine anomalies, endocrine dysfunction, auto immune disorders, maternal and paternal age, infectious diseases, environmental toxins, *etc*.

The incidence of balanced structural chromosomal abnormalities is 0.7% in the general population and increases to 2.2% after one miscarriage, 4.8% after 2 miscarriages and about 5.2% after 3 miscarriages (2). The balanced reciprocal translocations (BRTs) can be identified by conventional cytogenetic techniques but the cryptic or terminal translocations are difficult to identify.

Couples who have two or more miscarriages are at risk of structural chromosomal abnormality in one of the partners. Carriers of translocations of relatively small chromosomal regions onto an acrocentric short arm may have a very high risk of having unbalanced offspring (3, 4).

Thus, a larger short arm on acrocentric chromosomes warrants further studies in all cases to rule out a translocation especially if it is seen in a patient with intellectual disability or congenital anomalies or both (5). But in couples with bad obstetric history (BOH) cases, when there is a larger short arm, it is ideal to do an Ag-NOR staining to rule out any extra chromosomal material.

Chromosomal abnormalities, mainly BRTs, are common in couples with reproductive disorders including recurrent abortions (6). The presence of chromosomal rearrangements can lead to unequal crossing over during meiosis which can result in gametes with unbalanced chromosomes like duplications or deletions. Several different translocations involving almost all chromosomes are reported in BOH cases. Also, translocations involving chromosomes 11 and 13 are reported in literature. The translocations involving these chromosomes are mostly reported in cancers involving 11q23 region (7–10).

But there are no reports showing the apparently balanced reciprocal translocation involving these breakpoints associated with recurrent miscarriages involving chromosomes 11 and 13.

In this paper, the clinical cytogenetic and molecular cytogenetic findings in a patient with a balanced reciprocal translocation was reported involving chromosomes 11 and 13 identified by a simple silver staining technique. The characterization was done by other molecular cytogenetic techniques. To the best of our knowledge, this is the first report of a case presenting with translocation 11 and 13 chromosomes involving the unique breakpoints associated with RM.

## Case Presentation

### Subjects:

**Figure 1.**
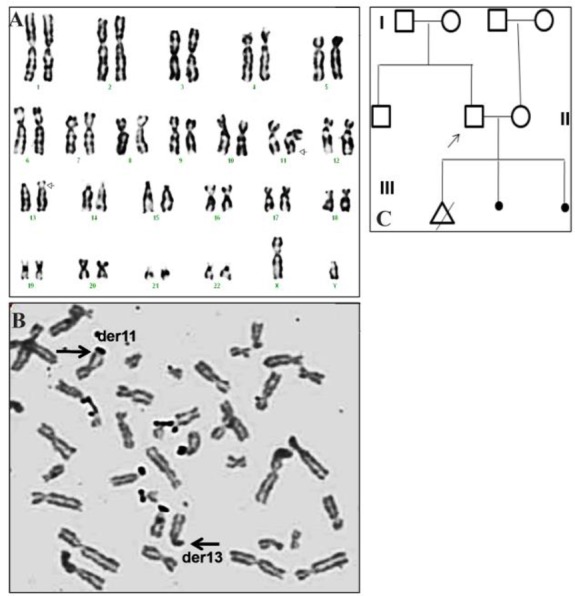
A: The GTG banded metaphase chromosomes of the patient showing 46,XY,13p+. B: NOR staining showing the black dots on all acrocentric chromosomes and on derivative 11q. No dots were seen on derivative 13p. C: The pedigree of the family is shown

A non-consanguineous couple, wife aged 25 years and husband 29 years was referred to our genetic clinic at Hyderabad, India for evaluation with a history of previous fetus having an additional material on p arm of chromosome 1 and also recurrent miscarriages in 2016. There was no history of any other disorders in the family. The detailed family history and written consent was taken from the couple. The pedigree of the family is also shown in figure 1C.

### Cytogenetic analyses:

Chromosomal analyses were carried out on the peripheral blood lymphocytes in the couple by standard methods. Metaphases were analyzed by GTG-banding using Trypsin and Giemsa, silver staining (NOR staining) which was also performed on the husband metaphase slides. The chromosomal analysis was also performed on the parents of the husband.

### Fluorescence in situ hybridization (FISH) analysis:

FISH was performed according to the manufacturer’s instructions on the metaphase spreads of the husband by standard procedures. Commercially available whole chromosome paint probe of chromosome 11 (Applied Spectral Imaging) and Acro-P-arm probe (Cytocell) were used. Further fine mapping was performed by selecting 15 BAC clones, 14 from chromosome 11 and 1 from chromosome 13 (Table 1) according to the current human NCBI Reference Sequence (11) utilizing the Ensembl and UCSC Genome browser (12). These clones were kindly provided by Dr. Vera Kalscheuer from the Max Planck Institute for Molecular Genetics, Berlin, Germany. The international standard nomenclature was used for clone names. BAC DNA was isolated using Nucleo-Bond Plasmid Midi kit (Macherey-Nagel, Dueren, Germany) according to manufacturer’s instructions. The isolated BAC DNA was labeled by Nick translation kit (Abbott Molecular, United States) using Red-dUTP and FISH experiments were performed on the patient metaphase slides as described by the standard protocols (13).

**Table 1.** BAC clones showing the FISH results on the derivative chromosomes

**S. No**	**BAC Clones**	**Chromosome band position**	**Position in the Genome (*bp*)**	**FISH signals**
**1.**	RP11-196I3	11q22.3	105430206–105571698	derivative 11
**2.**	RP11-447G4	11q23.3	116042124–116238529	derivative 11
**3.**	RP11-16M20	11q23.3	117191034–117367543	derivative 11
**4.**	RP11-158I9	11q23.3	118162404–118330697	derivative 13
**5.**	RP11-395H23	11q23.3	119108626–119251838	derivative 13
**6.**	RP11-89P5	11q23.3	120091053–120251056	derivative 13
**7.**	RP11-33N3	11q23.3–q24.1	121080589–121227945	derivative 13
**8.**	RP11-164B14	11q24.1	121790657–121946915	derivative 13
**9.**	RP11-10N17	11q24.2	124487812–124646260	derivative 13
**10.**	RP11-117F18	11q24.3	129292893–129465237	derivative 13
**11.**	RP11-151E22	11q25	133518752–133670501	derivative 13
**12.**	RP11-590N20	11q25	134010886–134204743	derivative 13
**13.**	CTD-2220K2	11q25	134340191–134436732	derivative 13
**14.**	RP11-269F1	11q13.5	75710374–75873742	derivative 11
**15**	RP11-77P19	13q11–q12.11	17918020–18079056	derivative 13

### Multiplex ligation-dependent probe amplification (MLPA):

MLPA was performed according to manufacturer’s recommendations (SALSA P036-E1, MRC-Holland). The P036-E1 human telomere-3 probe mix contains one MLPA probe for each subtelomeric region. Electrophoresis was performed using ABI 3100 Genetic analyzer (Applied Biosystems, USA) with ROX 500 size standard. Analysis of the raw data was done using GeneMarker software v1.85 (Applied Biosystems).

## Results

Chromosomal analysis of the wife showed 46, XX normal female karyotype, whereas the husband’s karyotype showed 46,XY,13p+ (Figure 1A), which was later refined to a de novo terminal translocation involving chromosomes 11 and 13. The parents of the husband showed normal karyo-type of 46,XX and 46,XY in the mother and the father. MLPA showed no copy number variations.

### Identification of the balanced reciprocal chromo-some:

In cases with an additional material on the short arm of the acrocentric chromosomes, silver staining was done to check the presence of satellites. The Nucleolar Organizing Regions (NOR) staining revealed satellites on the long arm terminal region of chromosome 11 and not on 13p region. Thus the terminal translocation involving chromosomes 11 and 13 was identified. In this case, NOR technique played a very important role in identifying the de novo balance reciprocal translocation (Figure 1B).

### Confirmation of the balance reciprocal translocation:

**Figure 2.**
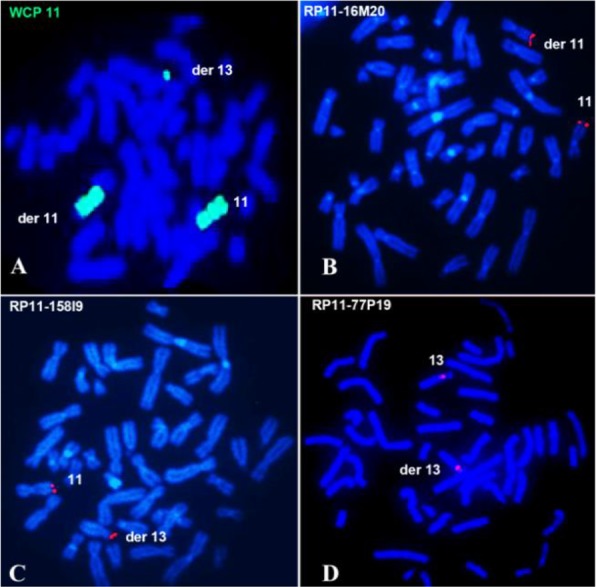
FISH on lymphocyte metaphase spreads of the patient. A: FISH with WCP 11 showing green signals on normal 11, derivative 11 and derivative 13 chromosomes. B: FISH with RP11-16M20 clone showing signals on normal chromosome 11 and derivative 11.C: FISH with RP11-158I9 clone showing signals on normal 11 and derivative 13.D: FISH with RP11-77P19 clone showing signals on normal chromosome 13 and derivative 13.

The balance reciprocal translocation was confirmed by whole chromosome FISH and Acro-P-Arm FISH probes and the short arm of the derivative 13 showed green signals confirming the chromosome 11 material (Figure 2A) and the Acro-P-Arm FISH showed signals on derivative chromosome 11 (Data not shown).

### Characterization of the balance reciprocal translocation:

**Figure 3.**
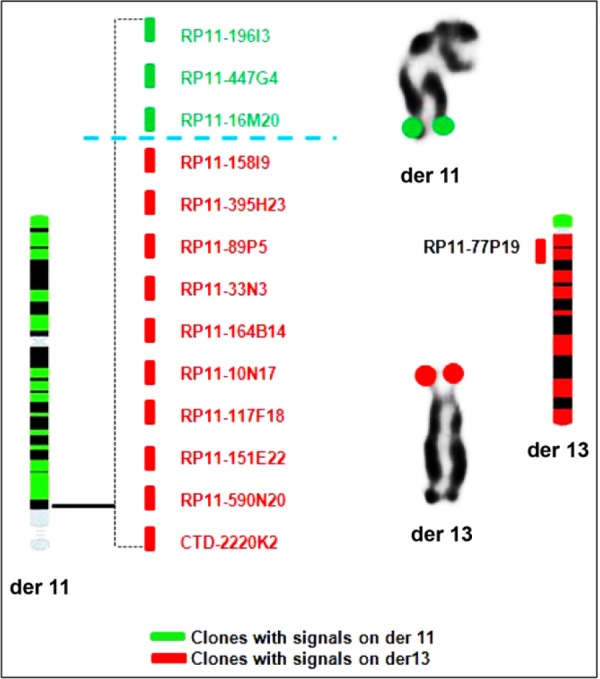
Physical map of the breakpoint regions of 11q23.2 and 13p region

The characterization of the balance reciprocal translocation was attained by BAC FISH. Fine mapping with the 14 BAC clones of chromo-some 11 showed the following results; four clones showed signals on normal 11 and on derivative 11 and the other 10 clones showed signals on normal chromosome 11 and derivative 13. Precisely, the breakpoint was identified between the two clones RP11-16M20 with signals on normal 11 and on derivative 11 (Figure 2B) and RP11-158I9 showed signals on normal chromosome 11 and derivative 13 (Figure 2C). The breakpoint was found to be within the region of 794 *kb* (Figure 3). Also, to identify the breakpoint on chromosome 13 region, one BAC clone RP11-77P19 covering the 13q11-13q12.11 region was selected and FISH with this clone showed signals on both the normal and derivative 13 chromosomes (Figure 2D), indicating that the breakpoint is only involving the p region and not the q region.

Hence the refined karyotype according to ISCN 2016 is 46,XY,t(11;13)(q23.3;p11.2).ish(wcp11+, RP11-196I3+,RP11-447G4+,RP11-16M20+, RP11-269F1+)(Acr-P-Arm+,RP11-158I9+,RP11-395H23+,RP11-89P5+,RP11-33N3+,RP11-164B14+,RP11-10N17+,RP11-117F18+,RP11-151E22+,RP11-590N20+,CTD-220K2+,RP11-77P19+).

## Discussion

BRT is a common chromosomal abnormality found in 1 in 500 people (14). It is indicated that 40% of the patients with apparently balanced translocations show a phenotype (15). Although no phenotypes are seen in the carriers of BRT they can give rise to reproductive problems causing pregnancy loss because of segregation during meiosis which results in gametes with duplication or deficiency of chromosome segments (16). A majority of miscarriages that occur before 10 weeks of gestation are due to chromosomal aneuploidies arising from new non-disjunction events; these events are more frequent in very early miscarriages (17).

A couple with RM was studied in this report. The karyotype in the husband showed an additional material on the acrocentric chromosome 13. The additional material was checked with NOR staining which ruled out the satellites and confirmed the euchromatic material. The satellites of chromosome 13 were found on the 11q region and the 11q material on 13p region and thus a BRT is identified which was also characterized. Hence, NOR played an important role in the identification of this terminal translocation. FISH mapping reduced the breakpoint region to 794 *Kb* on 11q region. In silico analysis of the breakpoint region in 11q revealed around 18 transcripts and genes but most of them are associated with acute lymphoid leukemias and acute myeloid leukemias and T cell receptors. There was only one gene TMP RSS4 (Transmembrane protease, Serine 4) which encodes a member of the serine family. Serine proteases are known to be involved in a variety of biological processes, whose malfunction often leads to human diseases and disorders. But this gene is also identified as an over expressed gene in pancreatic carcinoma.

Also, the DECIPHER (GRCh37) was checked for copy number variations in the breakpoint region and two duplicated regions, one with a 17.99 *Mb* region with a phenotype of abnormal heart morphology, diaphragm, nasal bridge, vasculature, cerebellar hemisphere hypoplasia and micrognathia were found. The second gain was a 28.42 *Mb* region with cryptorchidism, feeding difficulties in infancy, intellectual disability, microcephaly, micropenis, muscular hypotonia, obesity, short foot and short palm. The fetus might have received the derivative 13 from the father and thus a trisomy for the 11q23 region leading to the dosage sensitive genes in the region, leading to the death.

In the era of advanced molecular cytogenetic technologies, a simple NOR technique detected this terminal translocation. The reason of having a fetus with an additional material on chromosome 1 is independent of this BRT, but the miscarriages could be due to the trisomy of the 11q region.

Except conventional karyotyping, it is very difficult to identify the balanced reciprocal translocations in this era of developed molecular technologies too. The limitation of microarray is that it cannot detect the balanced translocations. MLPA also cannot detect the BRTs. Spectral karyotyping also cannot detect small terminal translocations. FISH is only a confirmatory technique to detect the balanced rearrangements. In cases involving translocations of acrocentric and autosomal chromosomes, NOR staining would help in the identification of terminal translocations.

In this study, no other technique could detect the translocation and without NOR, this translocation could be missed and it could be assumed as a normal polymorphic variant and the reason behind the recurrent miscarriages in the spouse could not be unfolded. As in couples with no other cause of miscarriages other than structural chromosomal rearrangements, nearly two-thirds are likely to have a normal outcome in subsequent pregnancy. The simple technique of NOR can be done in such cases to rule out any terminal translocations. Since the distal part of the long arm of chromo-some 11 is translocated to the tip of the short arm of chromosome 13, an unbalanced karyotype results in trisomy 11q without any other aneuploidy. This could define more precisely the clinical presentation of these chromosomal imbalances.

## Conclusion

In summary, the patient reported here increases our knowledge about the tests to be done for BOH couples. Without NOR, this translocation could be missed and a normal variant and the reason behind RM in the spouse could not be known. This helped us in proper counseling of the couple and advising the couple for prenatal diagnosis which would not be done in case of a variant identification.
